# Anaerobic gut fungi as biocatalysts: metabolic and physiological analysis of anaerobic gut fungi under diverse cultivation conditions

**DOI:** 10.3389/fmicb.2025.1662047

**Published:** 2025-09-19

**Authors:** Kevin Edward Schulz, Dominik Scholz, Anto Rafael Sikirić, Daniela Rambow, Anke Neumann, Katrin Ochsenreither

**Affiliations:** ^1^Institute of Process Engineering in Life Sciences 2: Electrobiotechnology, Karlsruhe Institute of Technology (KIT), Karlsruhe, Germany; ^2^Kaiserslautern University of Applied Sciences, Campus Pirmasens, Pirmasens, Germany

**Keywords:** morphology, particle size, wheat straw, growth form, metabolism, growth temperature, hydrogen

## Abstract

**Background:**

Anaerobic gut fungi, known for their diverse carbohydrate-active enzymes and hydrogen production, have promising potential for the valorization of lignocellulosic materials. Despite being classified nearly 50 years ago and re-categorized into the phylum *Neocallimastigomycota* in 2007, their growth conditions and metabolism remain largely underexplored. This study investigates the metabolic responses of *Aestipascuomyces dupliciliberans, Caecomyces churrovis, Khyollomyces ramosus, Orpinomyces joyonii, Pecoramyces ruminantium*, and *Neocallimastix cameroonii* under various conditions, including different growth temperatures, wheat straw particle sizes, alternative carbon sources, and cultivation methods.

**Results:**

Strain-specific differences were observed in temperature tolerance and metabolite production. Optimal growth occurred at 39 °C, while hydrogen production peaked at 41 °C in *N. cameroonii, P. ruminantium*, and *C. churrovis*. Larger wheat straw particles (2–3 mm) partially enhanced hydrogen yields, and soluble carbon sources such as glucose and cellobiose were efficiently metabolized, whereas xylose led to stress responses and low hydrogen output, particularly in *K. ramosus* and *O. joyonii*. High sugar concentrations triggered overflow metabolism, with increased lactate and formate production in *A. dupliciliberans* and *N. cameroonii*, while *K. ramosus*, lacking lactate dehydrogenase, accumulated formate and succinate. Fed-batch cultivation did not improve yields, likely due to substrate overfeeding and end-product inhibition. Biowaste substrates such as cucumber, carrot, and potato peels were effectively degraded and supported fungal growth. Notably, a novel morphological growth form was observed in *O. joyonii* under starvation conditions, suggesting a stress-induced developmental transition.

**Conclusion:**

This study provides valuable insights into the growth and physiology of anaerobic gut fungi and complements existing genomic data. The robustness of the process with respect to temperature, carbon source and substrate properties was evaluated, improving the understanding of anaerobic gut fungi cultivation and handling.

## Graphical Abstract

**Figure:**
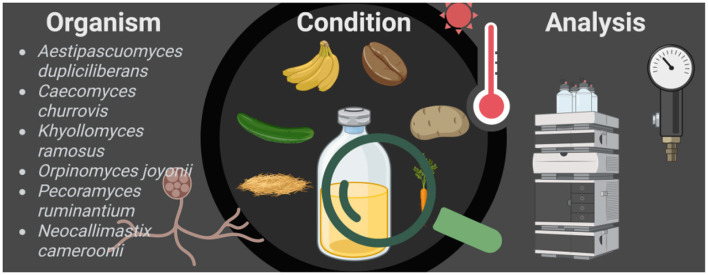

## Highlights

- Metabolic and physiological data of anaerobic gut fungi
- Effects of temperature on metabolism
- Particle sizes and fed-batch cultivation don't influence metabolism
- High potential in biowaste utilization
- New growth form of anaerobic gut fungi

## 1 Background

The steadily increasing global demand for platform chemicals, driven by globalization and population growthx, necessitates the development of renewable and readily available resources (Petricevic and Teece, 2019; Abel et al., 2016; Yang et al., 2022). Traditional fossil-based chemicals are not only finite but also contribute considerably to environmental pollution (De Guzman and Schiller, 2025; Wu et al., 2025). Consequently, a shift toward sustainable alternatives is essential. One promising approach is the utilization of lignocellulosic side streams derived from biowaste, such as residues from the paper and food industries, crop cultivation, and animal husbandry (Polizeli et al., 2005; Ringseis and Eder, 2025; Grossmann, 2024). These lignocellulosic materials could serve as a future base material for the production of various platform chemicals, which are vital for numerous industrial processes (Balchandani et al., 2025).

Current methods for processing lignocellulosic biomass often rely on harsh chemical pre-treatments that are both energy- and cost-intensive. Biological approaches, while more environmentally friendly, are typically uneconomical due to the high cost associated with enzyme treatments (Rahmati et al., 2020). A potential biotechnological solution lies in the use of evolutionarily adapted organisms.

Anaerobic gut fungi, which inhabit the digestive track of herbivores, have demonstrated exceptional efficiency in degrading lignocellulosic materials (Meili et al., 2023). Extracellular fungal cellulosomes—non-membrane-bound enzyme complexes composed of carbohydrate-binding domains and carbohydrate-active enzymes (CAZymes)—combined with mechanical penetration via appressorium-like structures, enable conversion rates of up to 48% for lignin-rich plant biomass (Ho et al., 1988; Jin et al., 2011; Hsin et al., 2025). The use of cellulosomes has been shown to be up to 12 times more effective than single-enzyme applications (Krauss et al., 2012). This underscores the potential of anaerobic fungi as biotechnological tools for efficient biomass conversion. However, effective application requires deeper understanding of their cultivation.

The phylum *Neocallimastigomycota* comprises strictly anaerobic fungi that play a key role in the digestion of plant material in herbivores (Hibbett et al., 2007). Despite being discovered nearly 50 years ago, optimal cultivation conditions for these fungi remain poorly understood, resulting in suboptimal cultivation practices (Orpin, 1977). Recent findings suggest that unique metabolites are produced in depending on the hosts species, indicating species-specific microbial interactions and metabolic profiles that depend on a balancedrumen microbiome (Omondi et al., 2024). Therefore, analyzing ecological parameters, growth conditions and media composition is essential for biotechnological applications such as biowaste utilization.

Anaerobic fungi generate energy through mixed-acid fermentation, which is linked to an incomplete citric acid cycle (Cheng et al., 2013; Song et al., 2009). The primary products—some of which are formed in hydrogenosomes, mitochondria-derived organelles—include acetate, formate, ethanol, lactate, hydrogen and succinate (Boxma et al., 2005; Marvin-Sikkema et al., 1990). While genetic and transcriptomic data offer insight into possible metabolic pathways, physiological studies remain scarce and are mostly limited to representatives of *Neocallimastix* and *Piromyces* (Wilken et al., 2021; Boxma et al., 2004). Building on the data and strains obtained and described by Stabel et al. (2021), this study aims to expand the understanding about carbon source utilization and process engineering for anaerobic gut fungi.

Therefore, the experimental design of this study followed a structured and progressive approach, beginning with the evaluation of the external environmental factor temperature, which is known to influence fungal growth and metabolism. Building on this, the impact of substrate particle size using the standard carbon source wheat straw. Subsequently, the metabolic responses to soluble carbon sources, including glucose, fructose, cellobiose, and xylose, was studied with additional consideration given to feeding strategies such as batch and fed-batch cultivation. Furthermore, biowaste-derived substrates were evaluated with regard to substrate biodegradability and metabolic output. Finally, this study concludes with the observation of a previously uncharacterized morphological growth form under starvation conditions, representing a novel finding with potential implications for fungal stress response and life cycle dynamics. This stepwise progression—from well-established parameters to exploratory observations—was designed to build upon existing knowledge while uncovering new insights into anaerobic fungal physiology.

## 2 Methods

### 2.1 Strains of anaerobic gut fungi

The anaerobic fungal strains used in this study, namely *Aestipascuomyces dupliciliberans* A252, *Caecomyces churrovis* PP313, *Khyollomyces ramosus* X2152, *Orpinomyces joyonii* W212, *Pecoramyces ruminantium* SA222, and *Neocallimastix cameroonii* G341, were isolated and characterized by Stabel et al. (2021), from various animal dung samples. The strains were cultivated in 7-day batch cycles.

### 2.2 Cultivation medium

The minimal medium used was based on the formulation by Stabel et al. (2021) with slight modifications.

The composition included per liter: 150 mL of salt solution 1 (3 g/L dipotassium hydrogen phosphate in demineralized water), 150 mL salt solution 2 (3 g/L potassium dihydrogen phosphate, 6 g/L ammonium sulfate, 6 g/L sodium chloride, 0.6 g/L magnesium sulfate heptahydrate, 0.6 g/L calcium chloride dihydrate in demineralised water), 2 mL of a zoospore inducer solution (0.5 g/L hemin chloride in 50% ethanol and 50% 0.05 M sodium hydroxide solution), 2 mL of a redox indicator solution (1 g/L resazurin sodium salt in demineralised water).

Demineralised water was added to a weight of either 990 g when a solid carbon source was used, or to 890 g if a soluble carbon source was added, depending on the experimental setup. Solid carbon sources were added directly to the serum flasks before autoclaving, while soluble carbon sources were sterilized separately and added after autoclaving the salt medium. The medium was mixed in Fernbach flasks, anaerobized with 100% CO_2._ The pH was adjusted to 6.9.

Subsequently, 6 g/L sodium hydrogen carbonate (buffer), 1 g/L L-cysteine hydrochloride monohydrate and 10 mL trace element solution (0.25 g/L manganese(II) chloride tetrahydrate, 0.25 g/L nickel(II) chloride hexahydrate, 0.25 g/L sodium molybdate dihydrate, 0.25 g/L boric acid, 0.2 g/L iron(II) sulfate heptahydrate, 0.05 g/L cobalt(II) chloride hexahydrate, 0.05 g/L sodium selenite pentahydrate, 0.05 g/L sodium metavanadate, 0.025 g/L zinc sulfate, and 0.0319 g/L copper(II) sulfate pentahydrate in 0.2 M hydrochloric acid) were added.

Unless otherwise stated, 1 mm sized wheat straw particles (Waldhof Qualitätsstroh, Landwirtschaftlicher Betrieb Uwe Hoferichter) were added as a solid carbon source to a final concentration of 5 g/L. The pH was adjusted using a 4 M NaOH solution. After aliquoting the medium into 50 mL (solid carbon source) or 45 ml (soluble carbon source) portions in 100 mL serum bottles, serum bottles were sealed with a butyl stopper and crimp caps, then autoclaved. Prior to inoculation, the medium is supplemented with 5 mL of the respective soluble carbon source from a 50 g/L stock solution resulting in a final concentration of 5 g/L, 0.5 mL of sterile vitamin solution (0.01 mg/L thiamine hydrochloride, 0.652 g/L panthothenic acid hemicalium salt, 0.6 g/L nicotinic acid, 0.02 g/L cyanocobalamin, 0.2 g/L biotin, 0.012 g/L pyridoxine hydrochloride, 0.05 g/L folic acid, and 0.05 g/L P-aminobenzoic acid 0.2 g/L riboflavin in demineralised water) and 0.5 mL antibiotic solution (50 g/L ampicillin sodium salt, 50 g/L streptomycin sulfate, 50 g/L penicillin sodium salt in demineralised water).

Strain maintenance was performed by weekly subculturing. Every 7 days, 5 mL of actively growing culture (inoculum) were transferred into a freshly prepared and fully supplemented serum bottle containing medium with 1 mm wheat straw as solid carbon source. This routine ensured continuous vitality and metabolic activity of all fungal strains under standardized conditions.

### 2.3 Experimental conditions

Under standard cultivation conditions, all fungal strains were maintained in serum bottles containing a minimal medium based on the formulation by Stabel et al. (2021), supplemented with wheat straw particles (1 mm, 5 g/L) as the solid carbon source. The medium was anaerobized with CO_2_, adjusted to pH 6.9, and enriched with trace elements, vitamins, and antibiotics. Cultures were incubated at 39 °C, and subcultured weekly to ensure consistent vitality.

To investigate the metabolic flexibility and environmental responsiveness of the strains, several key parameters were systematically varied. These included temperature, substrate particle size, carbon source type and concentration, feeding strategy, and the use of alternative biowaste materials. Each modification aimed to assess its impact on metabolite production, substrate utilization, and fungal morphology under controlled conditions

#### 2.3.1 Temperature screening

The medium was prepared with a solid carbon source (wheat straw) and inoculated with 5 mL inoculum of the respective strain. Cultivation was carried out in biological and technical triplicates over a period of 7 days at various incubation temperatures. The temperature range tested was from 33 to 44 °C in 2 °C increments, with additional measurements at 34 and 42 °C, to further investigate the boundaries of fungal growth. During cultivation, the incubator temperature was recorded daily using the external temperature sensor Pt100 GMH 3700 Series (Greisinger). Endpoint concentrations were analyzed for growth and the formation of gaseous and liquid metabolites as described in the Analysis section.

#### 2.3.2 Temperature shock

The medium was prepared with a solid carbon source (wheat straw). A preculture prepared for each strain was taken from the incubator and incubated at room temperature. Main cultures were inoculated with 5 mL of inoculum of the respective strain every 10 min over a period of 2 h, following the transfer of the pre-cultures to room temperature. Additionally, a final inoculation was performed after 24 h of incubation of the pre-culture at room temperature. Cultures were subsequently incubated at 39 °C for 7 days. This experiment was conducted as a single replicate. Analysis of the new generation was performed as described in the Analysis section.

#### 2.3.3 Substrate particle size

Wheat straw (Waldhof Qualitätsstroh, Landwirtschaftlicher Betrieb Uwe Hoferichter) was chopped to 2 mm and sieved into the following size fractions using standardized sieves (Retsch): <0.5 mm, 0.6–0.8 mm, 1–1.18 mm, 2–3 mm. These fractions were used as sole solid carbon source in the medium. Additionally, wheat straw was cut to 5 cm and also applied. Inoculation was carried out in duplicate with 5 mL of the respective culture. Cultivation was carried out for 7 days at the standard temperature of 39 °C as reported in literature (Stabel et al., 2021). Endpoint parameters were analyzed for growth and the formation of gaseous and liquid metabolites as described in the Analytics section.

#### 2.3.4 Usage of soluble carbon sources

To evaluate the influence of different initial soluble carbon source concentrations, salt media were supplemented after autoclaving to final concentrations of 1, 3, 5, 7, and 9 g/L of the soluble carbon sources glucose, fructose, xylose or cellobiose. Additionally, 5 g/L of 1 mm wheat straw particles were used as reference substrate. Inoculation was performed with 5 mL of the respective culture. The experiment was repeated three times. Cultivation was carried out for 7 days at 39 °C. Endpoint parameters were analyzed for growth and the formation of gaseous and liquid metabolites as described in the Analysis section.

#### 2.3.5 Evaluation batch vs. fed batch cultivation

A single addition of 5 mL sterile 50 g/L glucose solution was compared with a daily addition of 0.714 mL. Inoculation was performed with 5 mL of the respective culture. Incubation was carried out for 7 days at 39 °C. The experiment was carried out in biological and technical triplicates. Analysis was performed as described in the Analysis section.

#### 2.3.6 Biowaste valorization

To determine the potential of authentic biowaste streams with varying composition and degrees of lignification, reed, coffee grounds, organic cucumber peels, organic banana peels, organic carrot peels and organic potato peels were cut in small pieces and dried in the ED53 (WTCbinder) drying oven at 80 °C for 24 h. The cultivation medium was prepared differently than described above, using 5 g/L of each biowaste type as sole carbon source. Wheat straw was included as a reference substrate. Inoculation was performed with 5 mL of the respective culture and cultivation was carried out for 7 days at 39 °C. After cultivation the medium and residual carbon sources were transferred into pre-weighed 50 ml reaction tubes and dried in the ED53 (WTCbinder) at 80 °C until complete dry. The biomass was then weighed on the ADB 200-4 (Kern). The collected data was evaluated with the formular shown in Equation (1), where:

*a*: Percentage of biomass converted
*b*: Weight of the dried biomass + reaction tube
*c*: weight of the empty reaction tube
*d*: amount of biomass added to the bottle.

The experiment was conducted in technical triplicates. The weight of the fungal biomass was not accounted.


(1) $$\begin{array}{l} a=\left(\left(b-c\right)/\left(d\right)\right)\times 100 \end{array}$$

#### 2.3.7 Starvation experiment with *Orpinomyces joyonii*

In pre-conducted experiments, the growth of *O. joyonii* on glucose was considerably lower than on wheat straw. Therefore, starvation experiments were conducted on glucose. Cultivation was carried out in a two-step process. 5 mL of *Orpinomyces joyonii* pre-culture cultivated on wheat straw was used to inoculate medium with 5 g/L glucose as a carbon source. After incubation for 7 days at 39 °C, 5 mL of culture was transferred into a 2 L Schott bottle filled with 1 L medium containing 5 g/L glucose as a carbon source and again cultivated for 7 days at 39 °C. The resulting structures were photographed, sliced with a scalpel and examined under the microscope using the Eclipse E200 LED (Nikon Instruments Europe BV).

### 2.4 Analytics

All experiments were examined and analyzed using the techniques listed below, if not stated otherwise.

#### 2.4.1 Pressure measurement as vitality measurement

The gases formed during cultivation increase the pressure inside the inoculated serum bottles (Ochs Glasgeräte). The pressure was measured at the fermentation endpoint using either the vacuum gauge G 1111-VAC (Greisinger) and/or with the barometer GDH 14 AN (Greisinger) via neoprene tubes (Watson Marlow) using a Minisart NML 0.22 μm sterile filter (Sartorius) and a cannula (BD MicrolanceTM) by piercing the butyl stopper. The serum bottles were kept at cultivation temperature during measurement to avoid errors caused by temperature changes.

#### 2.4.2 Gas chromatography for metabolite analysis

The composition of the gas phase in the serum bottles was determined at the endpoint of the fermentation. Bottles were transferred to the water bath ONE 10 (Memmert GmbH + Co. KG) set to cultivation temperature. A gas sample of 3–5 mL was withdrawn and injected into the 3000 Micro GC System (INFICON Holding AG), which performed automatic sample intake at 10 mL/min using an internal vacuum pump. The sample inlet, the injection valve and the columns were operated at 80 °C. A two-channel analysis was used. For the quantification of hydrogen, nitrogen and oxygen, channel A was operated with a pre-column PLOTU column (Agilent Technologies), the main column Rt-Molsieve 5Å (Agilent Technologies) and helium as carrier and reference gas at 1.72 bar. The carbon dioxide concentration was analyzed via channel B with a Rt-Q-Bond column (Agilent Technologies) and 1.38 bar operating pressure. Detection was performed using a previously calibrated (using seven point calibrations) thermal conductivity detector (INFICON Holding AG).

#### 2.4.3 High performance liquid chromatography for metabolite analysis

Liquid metabolites were analyzed at the endpoint. A 2 mL sample was withdrawn from each of the serum bottles using a cannula (BD MicrolanceTM), transferred to a 2 mL reaction tube (Eppendorf) and centrifuged for 10 min at 13,000 rpm using a Heraeus TM Pico 17 (Thermo Fischer Scientific). One milliliter of the supernatant was filtered through a 0.22 μm sterile filter made of cellulose acetate (Restek) and transferred to a vial (Brown Chromatography Supplies), which was sealed with a Teflon cap. Analysis was performed using the 1100 HPLC system (Agilent Technologies). The pre-column used was the Rezex ROA organic acid H+ (8%) guard column (50 mm × 7.8 mm) (Phenomenex Ltd.), the main column a Rezex ROA organic acid H+ (8%) (300 mm × 7.8 mm, 8 m) (Phenomenex Ltd.), the eluent 5 mM sulphuric acid and the detectors RID (Agilent Technologies) and VWD (Agilent Technologies). The method ran for 40-min at a flow rate of 0.5 mL/min, with column and RID temperatures set to 50 °C and a VWD wavelength of 220 nm. Calibration was performed with the standards 0.1, 0.25, 0.5, 1, 2.5, 5, and 10 g/L of the chemicals acetate, ethanol, succinate, formate, lactate, malate, xylose, glucose and cellobiose.

## 3 Results

The effects of temperature, various solid and soluble carbon sources, carbon source concentrations and solid particle sizes on anaerobic fungal metabolism of six different species were analyzed. To evaluate metabolic activity, the total amount of metabolites produced—i.e., the final amount of organic acids, ethanol and hydrogen—was used as an aggregate parameter.

To minimize bias resulting from stochiometric differences among these compounds, carbon or electron balances have been proposed as a good practice. However, previous studies have shown that the total metabolite concentration serves as a reliable indicator of anaerobic fungal vitality, due to its strong correlation with overall metabolic enzyme activity (Stabel et al., 2022).

Production of single metabolites over time depending on strain and carbon source, i.e., temporal changes in the metabolic profile, are evaluated in Chapter 6.4.

### 3.1 Temperature screening

**Figure 1:**
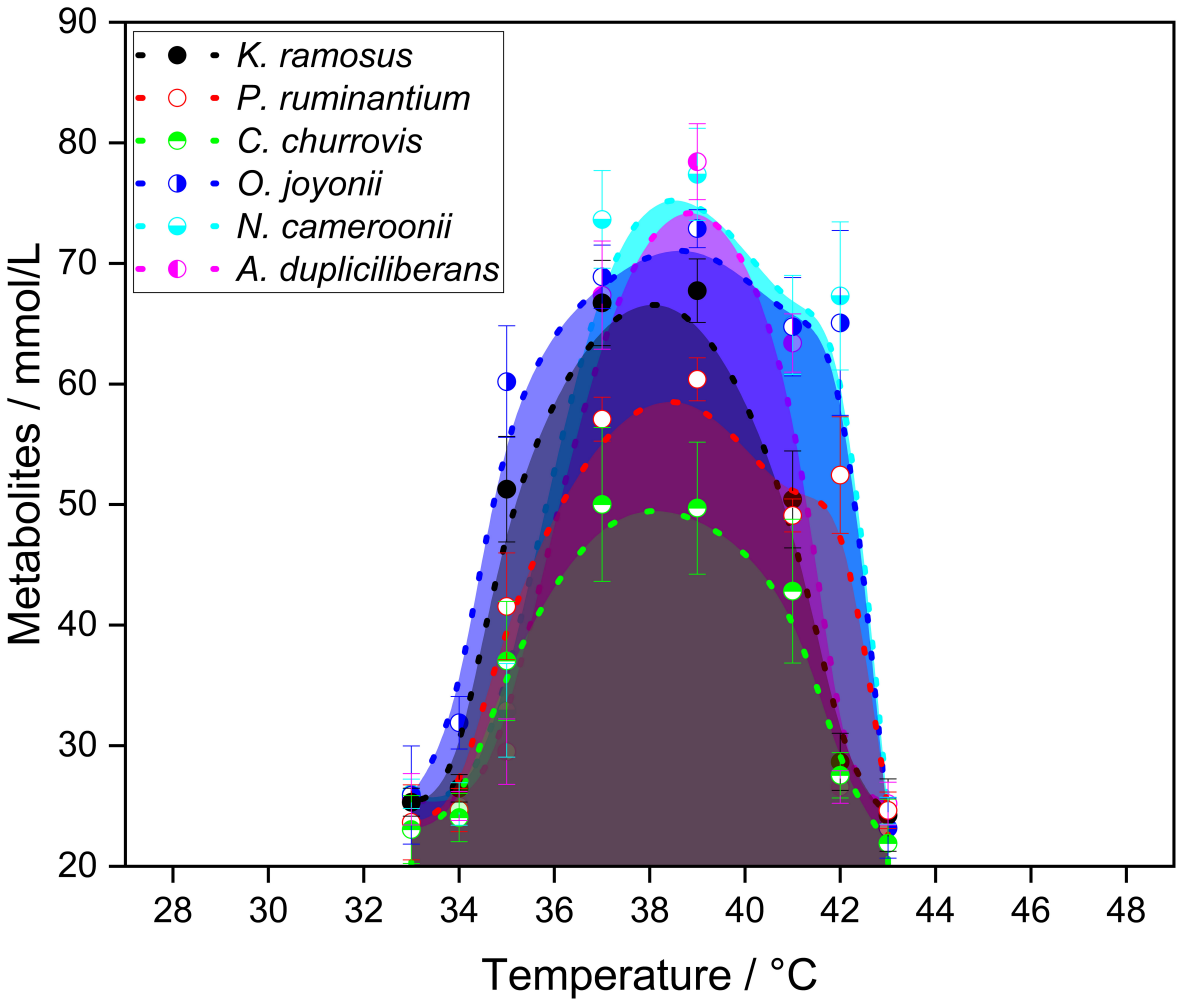
Total amount of metabolites produced after 7 days of cultivation of the tested strains in serum bottles with CO_2_ atmosphere and bicarbonate buffer on the carbon source wheat straw (n = 3).

As part of the basic characterization of the strains, their general growth temperature range and the optimal growth temperature were determined by cultivation over a wide temperature range using wheat straw as the carbon source. The total amounts of metabolites produced are presented in Figure 1, with a detailed breakdown shown in Appendix A, Supplementary Figure S1 and an overview provided in Supplementary Table S1.

All anaerobic gut fungi exhibited similar behavior across the tested temperature range. The general growth temperature range was defined by the minimum and maximum temperatures at which hydrogen production was still detectable. All strains grew at temperatures between 35 and 41 °C, with the exception of *A. dupliciliberans* A252 which showed a lower growth temperature limit of 37 °C.

Two temperatures were considered as candidates for the optimal growth temperature: one corresponding to the highest total metabolite production, and the other to the highest hydrogen yield. While most strains reached their peak metabolite production at 39 °C, *C. churrovis* showed the highest yield at 37 °C. In terms of hydrogen production, the optimal temperature varied among strains: 37 °C for *K. ramosus*, 39 °C for *C. churrovis*, and *A. dupliciliberans* and 41 °C for *P. ruminatium, O. joyonii*, and *N. cameroonii*, respectively.

### 3.2 Temperature shock

**Figure 2:**
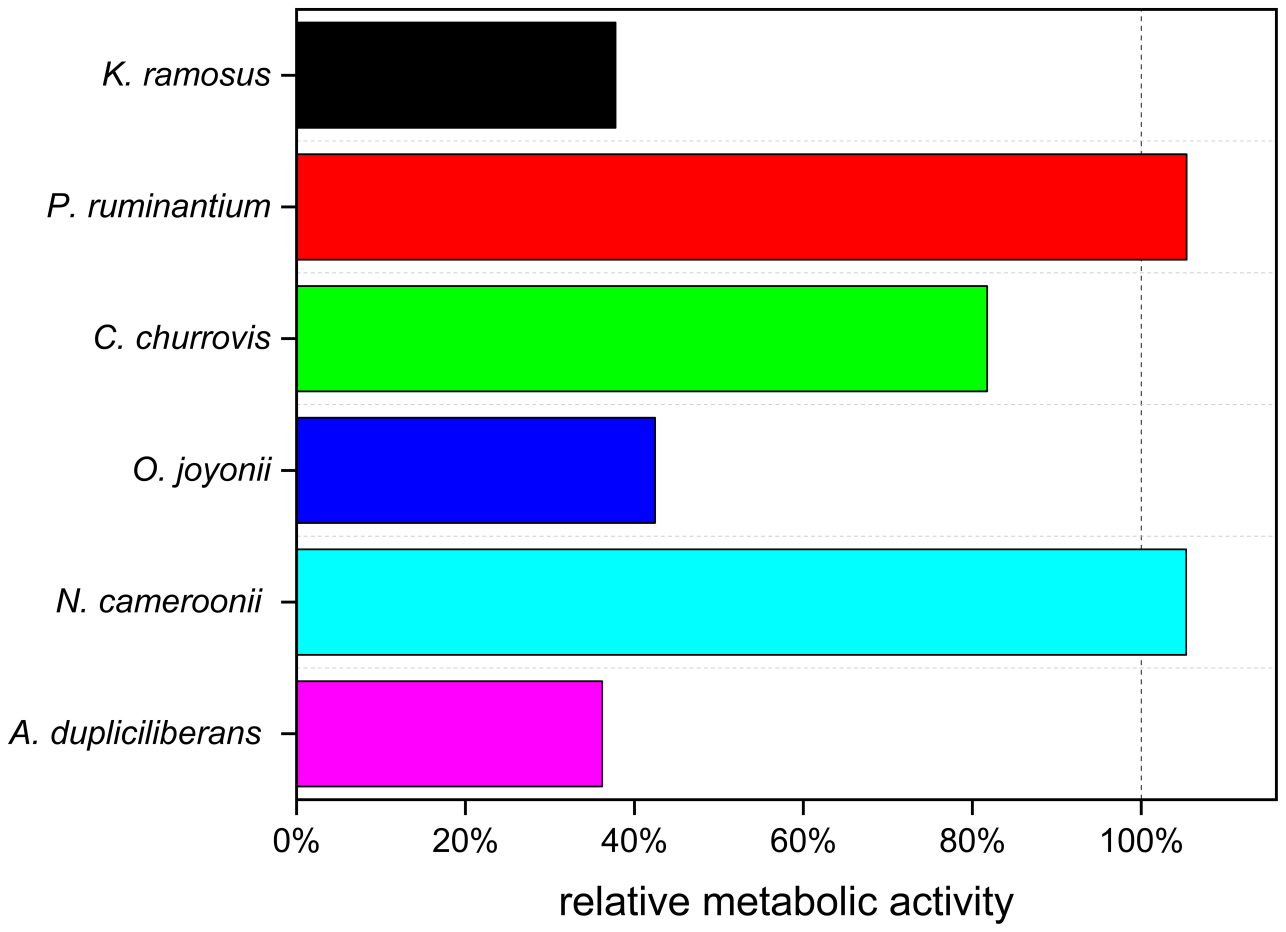
Effect of 24 h preculture room temperature shock to the metabolic activity of the next generation (*n* = 1).

Additionally, the resilience of anaerobic fungi to an induced room temperature shock was assessed by comparing the metabolic activity of the next generation of a non-shocked culture and shocked culture cultured on wheat straw as carbon source. Therefore, one culture of each strain was placed from 39 °C to room temperature and was used to inoculate subsequent cultures in 10-min intervals over 2 h and after 24 h. The inoculated cultures (next generation cultures) were cultivated at 39 °C. Figure 2 illustrates the relative metabolic activity of the next generation. A more detailed tracking of the first 2 h of the temperature exposure is provided in Appendix A, Supplementary Figure S2.

The treatment revealed varying sensitivities to the temperature shock. While next generation cultures of *K. ramosus* X2152, *O. joyonii* W212, and *A. dupliciliberans* A252 showed a considerable decrease in metabolic activity, the remaining three strains were largely unaffected.

### 3.3 Substrate particle size

**Figure 3:**
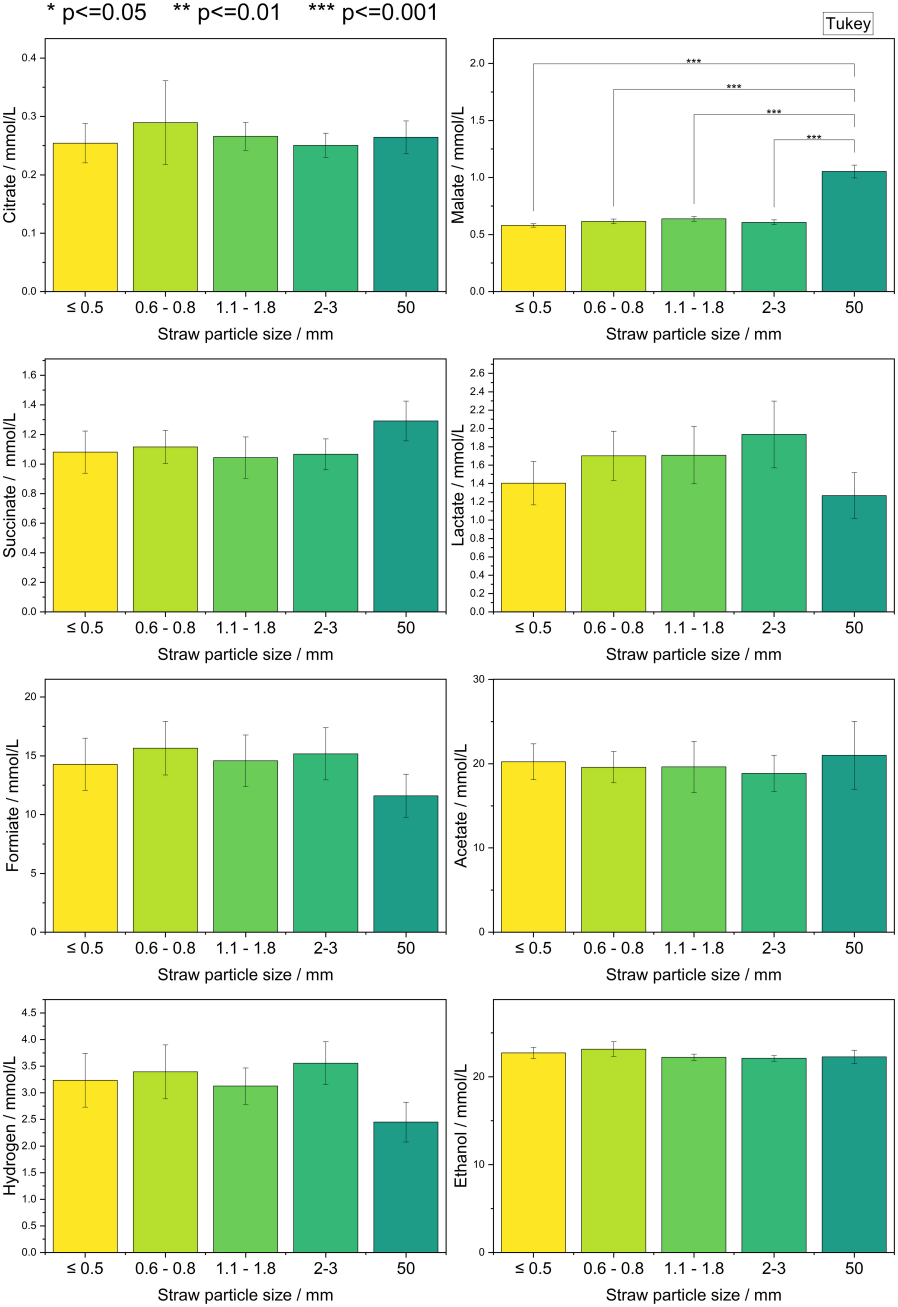
Statistical comparison for evaluation of significant differences in metabolite production based on particle size. All six tested strains are combined (*n* = 12).

Following the investigation of temperature as a process parameter, attention was directed toward the properties of the solid carbon source. Standard cultivation protocols for anaerobic fungi typically utilize lignocellulosic biomass, such as wheat straw, which is cut into small particles. In previous experiments, a particle size of 1 mm was used (Abbass et al., 2022). The metabolites produced by all tested anaerobic fungal strains were normalized and subsequently analyzed statistically using the Tukey test, as shown in Figure 3. Further statistical analysis is provided in Appendix A, Supplementary Section A5.1.

The only statistically significant correlation with *p* ≤ 0.001 observed was an increase in malate concentration associated with the largest particle size tested. No other significant differences in metabolite production were detected across the various substrate particle sizes. Absolute concentrations of the individual metabolites for each individual strain are presented in Appendix A, Supplementary Figure S3.

### 3.4 Usage of soluble carbon sources

**Figure 4:**
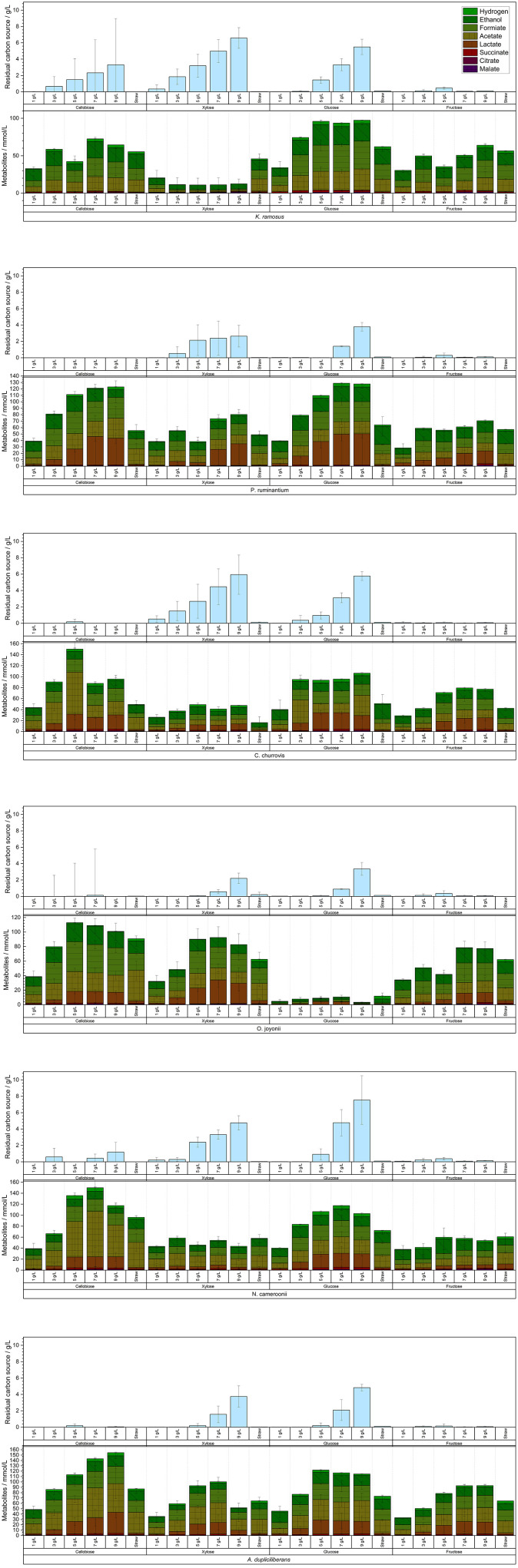
Produced metabolites with the different used soluble C-sources and residual concentration per experiment (*n* = 3).

While most anaerobic gut fungi are known to grow particularly well on solid lignocellulosic carbon sources, i.e., a complex substrate source, cultivation on defined soluble carbon sources offers the potential for deeper insights into their metabolic activity. The four soluble carbon sources selected for this study—glucose, xylose, fructose and cellobiose—were chosen based on the publication by Stabel et al. (2021) and applied over a wide initial concentration range. The quantities of metabolites produced by each tested strain, along with the residual concentrations of the carbon sources after 7 days of cultivation, are displayed in Figure 4.

**Figure 5:**
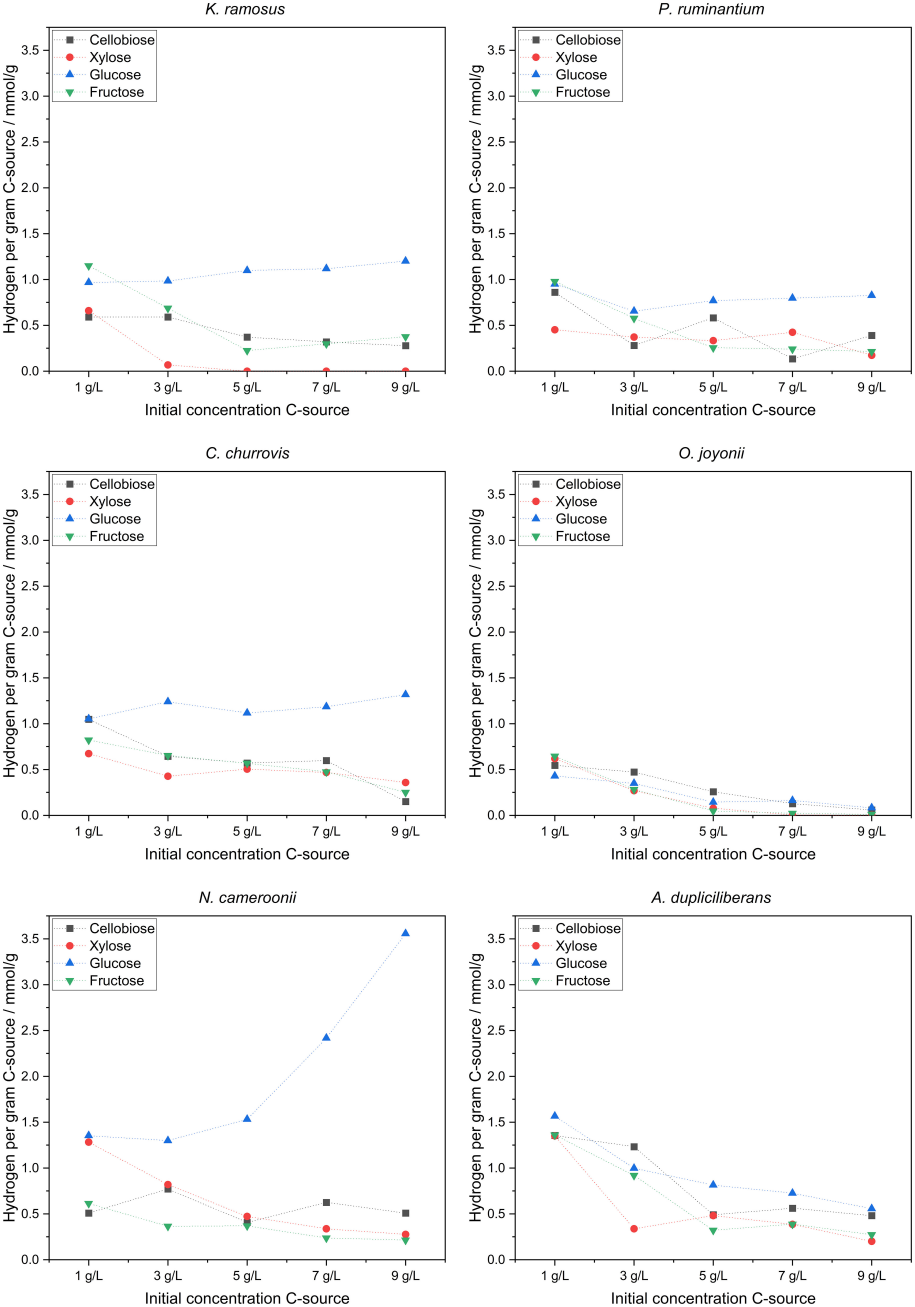
Hydrogen per gram carbon source produced.

With increasing initial carbon source concentration, a metabolic shift was detected—from hydrogen production toward elevated acetate and formate synthesis, eventually culminating in lactate production. Overall, this indicates a transition from hydrogenosomal to cytosolic metabolic pathways. Further analysis of hydrogen yields displayed in Figure 5, reveals a clear trend in the strains *N. cameroonii, C. churrovis*, and *K. ramosus*, with progressively higher hydrogen production observed at increasing carbon source concentrations.

**Figure 6:**
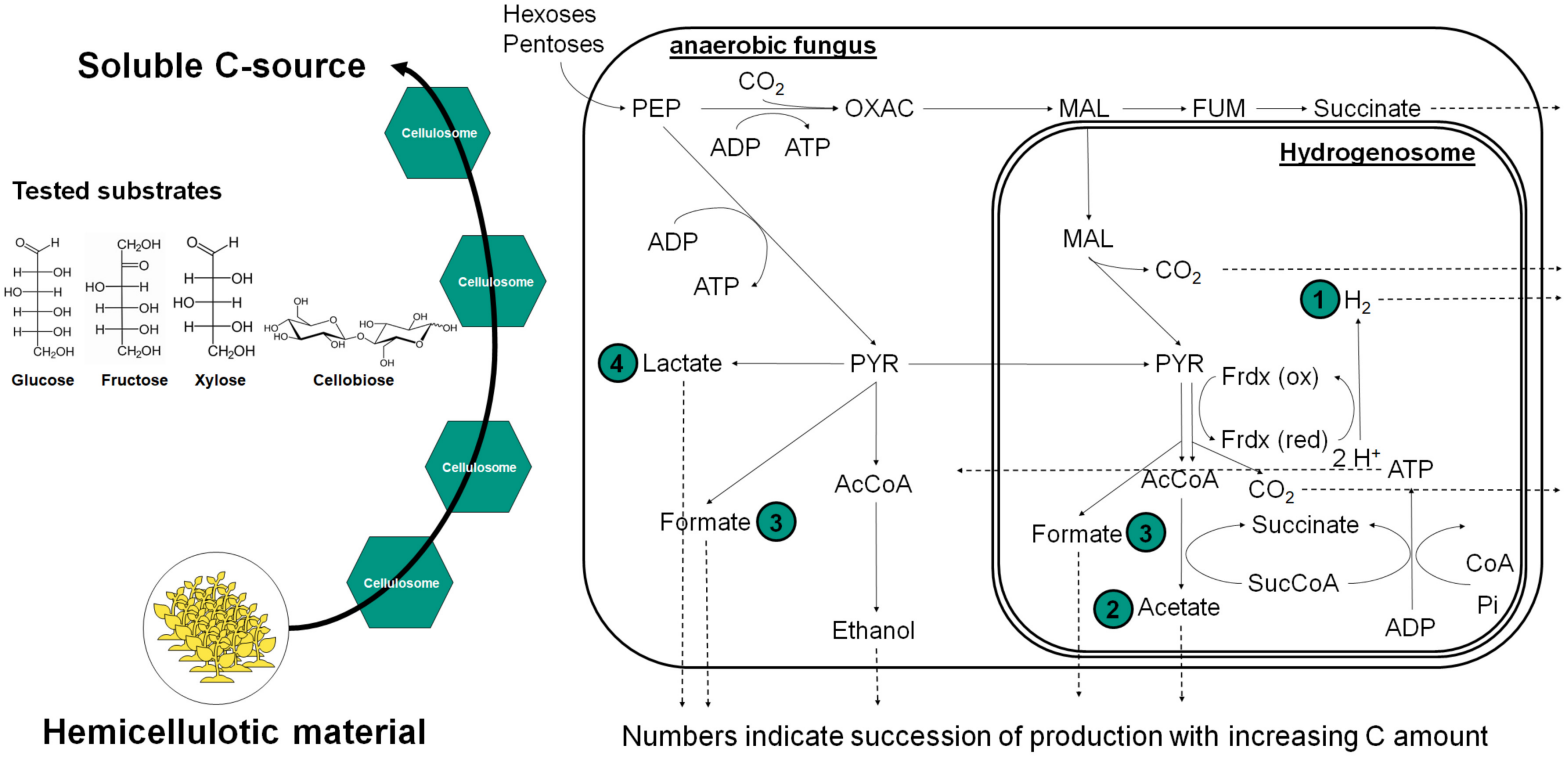
General overview of anaerobic gut fungi metabolic pathways and the succession in production of metabolites with increasing concentrations of soluble carbon source based on growth experiments with the six chosen fungal strains and data from Wilken et al. (2021).

Figure 6 gives an overview of the fungal metabolisms and summarizes the observed trends in the collected data.

### 3.5 Evaluation batch vs. fed batch

**Figure 7:**
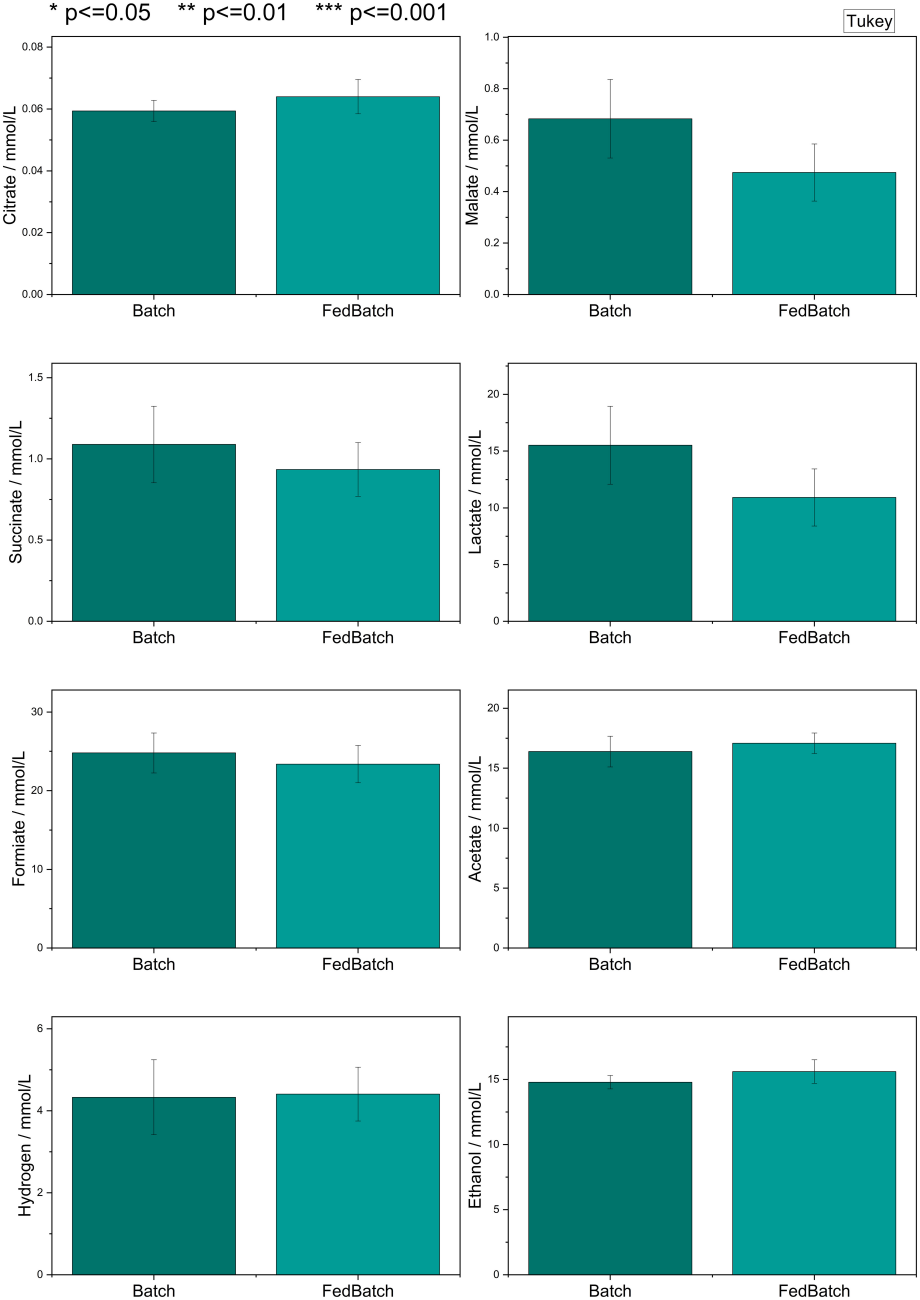
Statistical analysis of the produced metabolites after 7 days of incubation with the Tukey test for all conducted batch and fed batch fermentations in pairwise comparison (*n* = 18).

Following the evaluation of different carbon sources and initial carbon source concentrations, two feeding strategies were compared. In both tested approaches, the same amount of liquid carbon source (glucose) was used. In the batch fermentation, the entire amount was added at the start of cultivation. In contrast, the fed-batch fermentation involved daily additions of 1/7 of the total amount over a period of 7 days. Statistical analysis of the differences between the two strategies was performed using the Tukey test as displayed in Figure 7. A detailed analysis of the produced metabolites per strain is displayed in Appendix A, Supplementary Figure S4. Further statistical analysis is provided in Appendix A, Supplementary Section A5.2.

No statistically significant differences with *p* ≤ 0.05 in the absolute metabolite production were observed between the two feeding strategies across all tested strains.

### 3.6 Biowaste valorization

**Figure 8:**
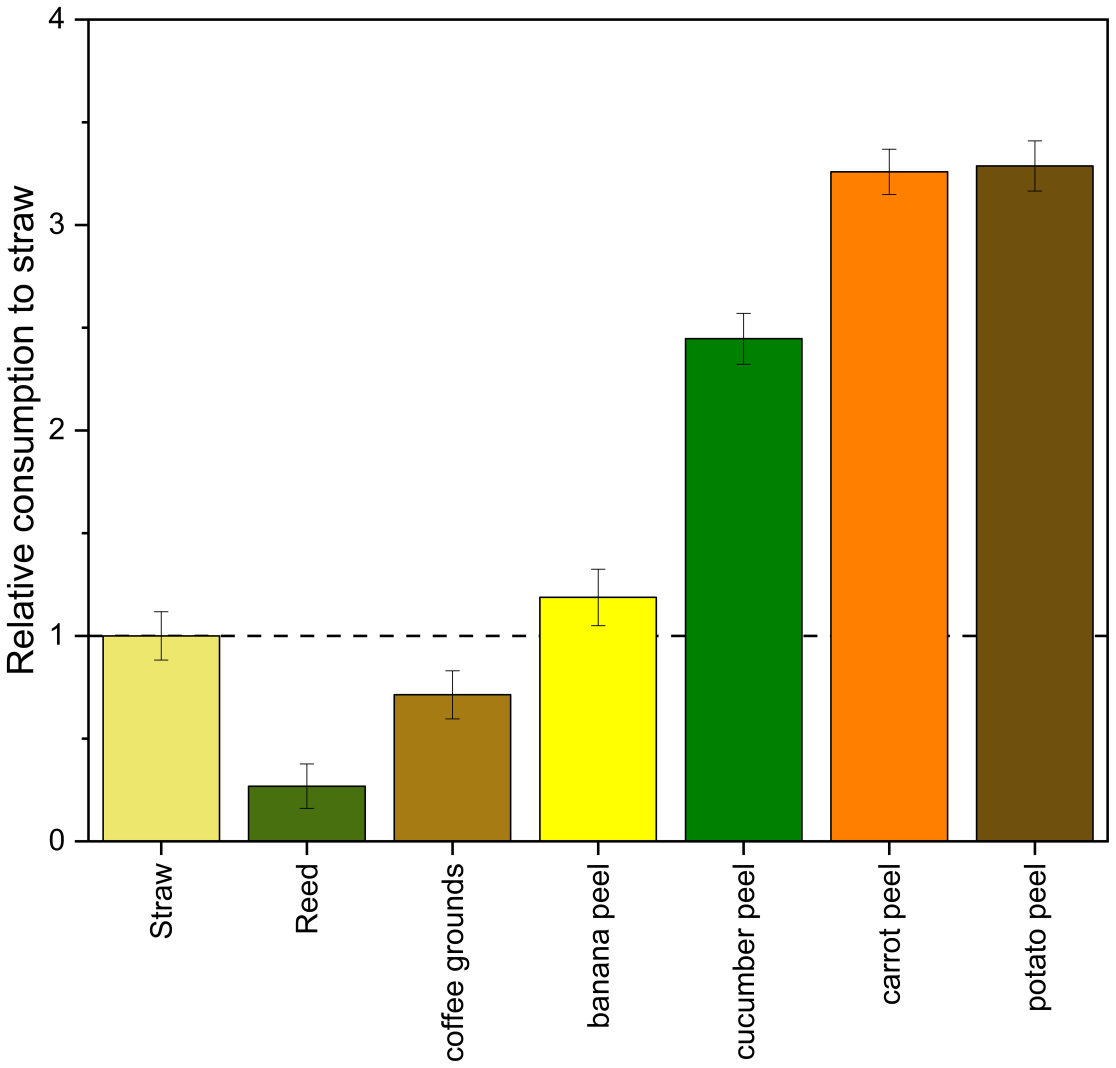
Relative consumption of solid carbon sources tested in comparison to wheat straw (*n* = 3).

Based on the results presented above, further questions arose regarding the biotechnological potential of anaerobic fungi, particularly in relation to their versatility in the valorization of various biowastes. To evaluate this potential, the mass of solid carbon source consumed during cultivation—using reed, coffee grounds, organic cucumber peels, organic banana peels, organic carrot peels and organic potato peels—was determined and compared to wheat straw as reference substrate. Relative consumption values, calculated from the total metabolite production in comparison to wheat straw cultivation, are illustrated in Figure 8.

Banana peels, cucumber peels, carrot peels, and potato peels were consumed to a greater extent than wheat straw, indicating higher biodegradability and a promising potential for biowaste valorization. In contrast, reed and coffee grounds exhibited lower consumption rates, suggesting limited suitability as substrates. These findings highlight the promising potential of selected kitchen and environmental waste materials as alternative substrates for anaerobic fungal cultivation.

### 3.7 Starvation experiment with *Orpinomyces joyonii*

**Figure 9:**
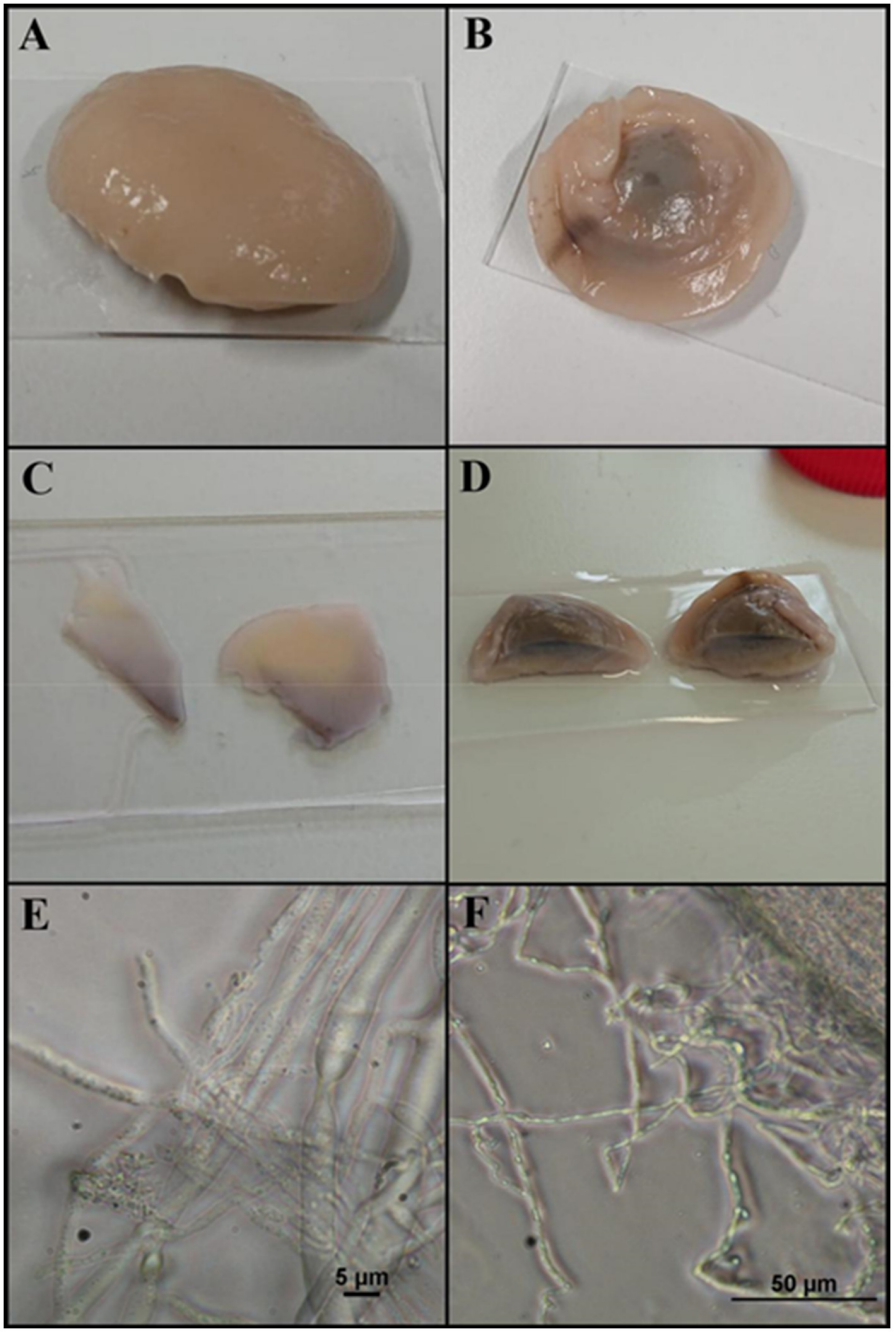
New growth form of O. joyonii emerged after prolonged cultivation on the soluble carbon source glucose. For comparison a standard slide with 26 mm × 76 mm was used. **(A)** View from above, small holes/channels are visible; **(B)** view from the bottom; **(C)** slices of the biomass showing a shift in color with the bright sight showing the upper side and the purple side the lower side; **(D)** biomass cut in half; **(E,F)** microscopic pictures of the structure in bright field.

Finally, the behavior of *O. joyonii* strain W212 under nutrient-limitation was investigated. This strain had previously shown the highest dependency on solid carbon sources in unpublished pre-experiments—a trend also reflected in the results presented in Figure 5. During prolonged cultivation on a soluble carbon source, a novel growth form emerged. This morphology was documented photographically and is shown in Figure 9. The observed form was consistently reproduced in independent cultivations (data not shown).

## 4 Discussion

To comprehensively assess the metabolic versatility of the investigated strains, a broad screening was conducted to evaluate their ability to utilize a variety of carbon sources. Effective assimilation of these substrates depends not only on the presence of the required genetic and enzymatic machinery, but also on the environmental conditions under which these pathways are activated. Among these, temperature plays a critical role, as enzymatic activity is inherently temperature-dependent (McLeod, 2025). To account for this, temperature screening experiments were performed across a range of conditions. Although all strains exhibited comparable tolerance ranges, distinct differences in their optimal growth temperatures were observed, indicating strain-specific thermal preferences.

In addition to identifying optimal growth conditions, the robustness of the strains under suboptimal—particularly cold—conditions was evaluated. *A. dupliciliberans, C. churrovis*, and *N. cameroonii* exhibited enhanced thermotolerance and resilience to cold exposure, which is advantageous for industrial applications. This increased robustness facilitates easier handling, storage, and transportation, thereby reducing logistical constraints and associated costs (Matsushita et al., 2016).

Another critical factor in the biotechnological application of lignocellulosic biomass is the pretreatment of the selected carbon sources. Such processes are often energy-intensive and economically challenging (Rahmati et al., 2020). To investigate whether substrate particle size influences fungal metabolism, substrates of varying sizes were tested. Interestingly, no significant changes in the overall metabolic profiles of the anaerobic fungi were observed, except for a notable increase in malate concentration when particles of 50 mm were used.

Since malate is an intracellular metabolite, its release may indicate cell lysis or metabolic stress. In filamentous fungi such as *Mucor circinelloides*, malate transport is closely linked to metabolic reorganization under stress conditions (Chen et al., 2024). It is therefore conceivable that exposure to large particle sizes—which may limit nutrient availability—leads to increased malate release, potentially as a result of prolonged stress or nutrient limitation (Zhao et al., 2018). This delayed substrate utilization suggests an extended adaptation or starvation phase prior to the onset of active metabolism. These findings imply that, under certain conditions, extensive pretreatment may not be necessary—particularly in large-scale fermentations. Reducing the degree of pretreatment could significantly lower operational costs without compromising metabolic efficiency (Pasalari et al., 2024).

Although solid carbon sources are traditionally used for the cultivation of anaerobic fungi, soluble carbon sources offer several notable advantages. These include improved dosing accuracy, enhanced bioavailability, and the potential use of inexpensive side streams like glucose syrup (Yang et al., 2024; Yaseen et al., 2013). In addition, soluble substrates provide logistical benefits, including reduced transportation costs due to their higher packing density and easier handling in automated systems (Schoemaker et al., 2003). Based on the findings from Stabel et al. (2021) the soluble carbon sources glucose, fructose, cellobiose and xylose were investigated.

Anaerobic fungi of the phylum *Neocallimastigomycota* exhibit pronounced metabolic flexibility in response to both type and concentration of carbon sources, with significant implications for hydrogen production and metabolite profiles. In the strains *N. cameroonii, C. churrovis*, and *K. ramosus*, increasing concentrations of glucose led to elevated hydrogen yields per gram of substrate, despite a reduction in substrate uptake efficiency. Hydrogenosomal metabolism—typically associated with acetate and hydrogen production—was dominant at lower substrate concentrations but declined as cytosolic pathways became more active. The bifurcating hydrogenase proposed by Wilken et al. (2021) may explain the sustained hydrogen output even under high glucose conditions. Overall, this decoupling of substrate uptake and product yield suggests a shift toward overflow metabolism, in which excess pyruvate is redirected from hydrogenosomal pathways to cytosolic mixed-acid fermentation. This results in increased production of lactate, formate, and succinate, mirroring metabolic phenomena such as the Crabtree and Warburg effects observed in other organisms (Battjes et al., 2024). Notably, *A. dupliciliberans* and *N. cameroonii* showed increased lactate production at higher sugar concentrations, while *K. ramosus*, lacking lactate dehydrogenase activity, favored the accumulation of formate and succinate. The presence of malate and citrate in the extracellular medium—particularly under xylose-rich conditions—indicates cellular lysis and metabolic stress, especially in strains such as *K. ramosus* and *O. joyonii*.

Cellobiose and glucose emerged as the most efficiently metabolized substrates across nearly all strains, likely due to the fungi's robust cellulolytic enzyme systems, including CAZymes and cellulosomes. In contrast, xylose was poorly utilized by most strains, as evidenced by high residual concentrations and low hydrogen yields. An exception was *O. joyonii*, which demonstrated efficient xylose uptake but limited conversion to hydrogen, further supporting a shift toward non-hydrogenogenic metabolic pathways.

Overall, these findings highlight a dynamic reprogramming of central carbon metabolism in anaerobic fungi, driven by substrate availability and concentration. The transition from hydrogenosomal to cytosolic fermentation under substrate-rich conditions reflects a trade-off between energy efficiency and metabolic throughput. This metabolic shift has important implications for optimizing biotechnological processes such as biohydrogen production and lignocellulosic biomass conversion.

An additional critical parameter for optimizing the production of target metabolites in anaerobic fungal fermentations is the applied feeding strategy. To assess its impact, conventional batch and fed-batch cultivation modes were compared in terms of metabolic output. However, the transition from batch to fed-batch cultivation did not result in any statistically significant improvement in metabolite production. This outcome suggests two potential limiting factors: first, the likelihood of substrate overfeeding in the fed-batch setup, which may have led to metabolic saturation or stress responses; and second, the accumulation of inhibitory end-products, such as organic acids, ethanol or hydrogen—which can negatively affect cellular metabolism and growth.

These findings indicate that simply increasing substrate availability through fed-batch operation does not necessarily lead to higher metabolite yields. Instead, they underscore the importance of carefully balancing substrate input with the metabolic capacity of the organism, as well as mitigating the accumulation of inhibitory by-products. Future optimization efforts should therefore consider dynamic feeding strategies—such as feedback-controlled feeding—in combination with *in situ* product removal or pH control. These approaches can help maintain favorable metabolic conditions and prevent pathway inhibition, thereby enhancing overall process efficiency.

In addition to soluble carbon sources, biowaste materials were considered for further improvement. Comparative analysis revealed that banana peels, cucumber peels, carrot peels, and potato peels exhibited higher consumption rates than wheat straw, indicating superior biodegradability and enhanced suitability as substrates for anaerobic fungal cultivation. These materials likely provide more accessible carbon sources and fewer structural barriers, making them promising candidates for biowaste valorization in fungal bioprocesses (Nguyen and Nguyen, 2023; Kalogiannis et al., 2023). In contrast, reed and coffee grounds showed lower degradation levels, suggesting limited applicability due to their recalcitrant composition or potential inhibitory compounds (Czubaszek and Wysocka-Czubaszek, 2025; Hernandez-Hosaka et al., 2024). Overall, these findings underscore the potential of selected kitchen waste streams as sustainable and efficient alternatives to conventional lignocellulosic feedstocks in anaerobic fungal biotechnology.

A previously uncharacterized growth form was observed in strain *O. joyonii*, representing a potentially impactful discovery in the life cycle or stress response of anaerobic fungi. Preliminary observations suggest that this morphological shift is induced under conditions of prolonged fiber limitation and sustained environmental stress. The applied cultivation method, which involved gradual starvation, appears to have triggered this transformation. A comparable phenomenon was reported by Stabel et al. (2022) who documented the formation of fungal pellets during fermentation, indicating that morphological plasticity may be a common response to adverse conditions.

The nature of this newly observed form remains to be elucidated, but its emergence under stress conditions raises the possibility that it may represent a survival structure or even a sexual reproductive stage. Intriguingly, the formation of such structures may also be influenced by mechanical stimuli, as suggested by the experimental setup from Stabel et al. (2022). If this hypothesis holds true, it could imply that mechanical forces encountered during gut passage—such as peristalsis or abrasive interactions with digesta—may play a role in triggering developmental transitions. This would have profound implications for understanding the ecology and transmission of anaerobic gut fungi within herbivorous hosts, potentially revealing a mechanism by which fungal propagules are activated or dispersed during host-to-host transfer.

Further investigation, including microscopic characterization, transcriptomic profiling, and controlled mechanical stimulation experiments, will be essential to elucidate the biological function and regulatory mechanisms underlying this novel growth form.

## 5 Conclusion

This study provides several novel insights into the metabolic behavior and biotechnological potential of anaerobic gut fungi that go beyond the scope of previous research. While earlier studies such as Stabel et al. (2021) focused on the biochemical characterization of individual strains and their metabolite profiles under standard conditions, and Wilken et al. (2021) explored genomic adaptations like horizontal gene transfer, and Omondi et al. (2024) offered a comparative multi-omics perspective across host species, the present work delivers a systematic, multifactorial analysis of fungal metabolism under varying environmental and nutritional conditions using six distinct strains. One of the most significant contributions is the discovery of a previously uncharacterized growth form in *O. joyonii* under starvation conditions, which may represent a survival or reproductive stage. This morphological shift, potentially triggered by mechanical stimuli, introduces a new dimension to the ecological understanding of anaerobic fungi and their transmission within herbivorous hosts. Furthermore, the study demonstrates a concentration-dependent metabolic reprogramming, where increasing substrate availability leads to a shift from hydrogenosomal to cytosolic fermentation pathways. This shift is characterized by increased production of lactate, formate, and succinate, and reflects overflow metabolism similar to the Crabtree and Warburg effects observed in other organisms. The sustained hydrogen production under high glucose conditions, potentially mediated by bifurcating hydrogenase activity as proposed by Wilken et al., highlights the metabolic flexibility of these fungi. In addition, the work evaluates the biodegradability of various kitchen waste materials—such as banana, carrot, and potato peels—revealing them as superior substrates compared to wheat straw, which has direct implications for sustainable feedstock selection in industrial applications. Unlike previous studies that focused primarily on lignocellulosic substrates, this research expands the substrate spectrum and demonstrates the fungi's capacity to valorize diverse biowaste streams. Another important finding is that fed-batch cultivation does not improve metabolite yields, likely due to overfeeding and end-product inhibition, emphasizing the need for adaptive feeding strategies and *in situ* product removal. Moreover, the observation that substrate particle size has minimal impact on overall metabolite production suggests that costly pretreatment steps may be unnecessary, offering economic advantages for scale-up. Taken together, these findings significantly advance the current understanding of anaerobic fungal metabolism and provide actionable insights for optimizing biotechnological processes, from substrate selection to fermentation strategy and ecological interpretation.

To further expand the understanding of anaerobic fungal metabolism and its biotechnological potential, we propose several additional experiments. First, pH-dependent cultivation trials should be designed to investigate how varying acidity levels influence metabolic fluxes and product distribution, particularly the balance between hydrogenosomal and cytosolic fermentation. Second, long-term cultivation experiments should be initiated to assess adaptive responses over multiple generations, potentially revealing strain-specific improvements in substrate utilization and stress tolerance, and also showing limitations in the current cultivation strategy. Additionally, co-feeding strategies combining soluble sugars with biowaste-derived substrates should be tested to evaluate synergistic effects on metabolic output and substrate degradation efficiency. To mitigate end-product inhibition, *in situ* product removal techniques—such as membrane filtration and adsorptive resins—should be explored, aiming to enhance metabolite yields by maintaining favorable fermentation conditions in serum bottles. Mechanical stimulation experiments should also be conducted to simulate peristaltic forces and assess their role in triggering morphological transitions, including pellet formation or stress-induced growth forms. Finally, mixed-strain cultures should be evaluated to determine whether interspecies interactions could promote complementary metabolic pathways and improve overall process performance.

## Data Availability

The raw data supporting the conclusions of this article will be made available by the authors, without undue reservation.
