# Supplementary material for: Anaerobic gut fungi as biocatalysts: metabolic and physiological analysis of anaerobic gut fungi under diverse cultivation conditions
Source: Front Microbiol. 2025 Sep 19;16:1662047. doi: 10.3389/fmicb.2025.1662047 (PMC12492954; doi:10.3389/fmicb.2025.1662047)
Supplement: Supplementary file 1 [file Supplementary_file_1.docx]

| Organism | **Growth temperature range / °C** | Optimum temperature metabolites / °C | Most metabolites produced / mmol/L | Optimum temperature hydrogen / °C | Hydrogen produced / mmol/L | Hydrogen mmol / g straw |
| --- | --- | --- | --- | --- | --- | --- |
| *K. ramosus* | 35 - 41 | 39 | 68 ± 05 | 37 | 4.10 ± 0.29 | 0.82 ± 0.06 |
| *P. ruminantium* | 35 - 42 | 39 | 60 ± 03 | 41 | 2.56 ± 0.16 | 0.51 ± 0.03 |
| *C. churrovis* | 35 - 41 | 37 | 50 ± 09 | 39 | 2.36 ± 0.31 | 0.47 ± 0.06 |
| *O. joyonii* | 35 - 42 | 39 | 73 ± 03 | 41 | 4.65 ± 0.58 | 0.93 ± 0.12 |
| *N. cameroonii* | 35 - 42 | 39 | 77 ± 08 | 41 | 4.37 ± 0.36 | 0.87 ± 0.07 |
| *A. dupliciliberans* | 37 - 41 | 39 | 78 ± 06 | 39 | 6.54 ± 0.24 | 1.31 ± 0.05 |

**Appendix A Supplementary material**

**A1. Temperature screening**

*Table S1: Overview of gathered data from the temperature screening. Growth temperature range is* *defined by the minimal and maximal temperature where hydrogen was produced.*





Figure S1: Metabolites produced at different temperatures for the tested strains.

**A2. Temperature shock**





Figure S2: Relative metabolic activity of the next generation after temperature shock at room temperature.

**A3. Substrate particle size**





Figure S3: Total metabolite amount produced with different substrate particle sizes.

**A4. Evaluation batch vs. fed batch cultivation**

**

**

Figure S4: Amount of produced metabolites in batch and fed batch approach.

**A5. Further statistical analysis**

**A5.1 Substrate particle size**

Table S2: Mean values, standard deviation, standard error of the mean and 95 % confidance intervals from the conducted tukey test for particle sizes.

| Substrate | Particle size | Mean value | Standard deviation | Standard Error of the Mean | 95 % -Confidence  Intervals  Lower boundary | 95 % -Confidence  Intervals  Upper boundary |
| --- | --- | --- | --- | --- | --- | --- |
| Citrate | ≤ 0.5 | 0.25417 | 0.08246 | 0.03367 | 0.1881768 | 0.3201632 |
|  | 0.6 - 0.8 | 0.28947 | 0.17579 | 0.07177 | 0.1488008 | 0.4301392 |
|  | 1.1 - 1.8 | 0.26607 | 0.05896 | 0.02407 | 0.2188928 | 0.3132472 |
|  | 2-3 | 0.25057 | 0.05081 | 0.02074 | 0.2099196 | 0.2912204 |
|  | 50 | 0.2643 | 0.06906 | 0.02819 | 0.2090476 | 0.3195524 |
| Malate | ≤ 0.5 | 0.57963 | 0.03453 | 0.0141 | 0.551994 | 0.607266 |
|  | 0.6 - 0.8 | 0.6154 | 0.04983 | 0.02034 | 0.5755336 | 0.6552664 |
|  | 1.1 - 1.8 | 0.63677 | 0.05183 | 0.02116 | 0.5952964 | 0.6782436 |
|  | 2-3 | 0.60723 | 0.05321 | 0.02172 | 0.5646588 | 0.6498012 |
|  | 50 | 1.0531 | 0.13715 | 0.05599 | 0.9433596 | 1.1628404 |
| Succinate | ≤ 0.5 | 1.0811 | 0.34931 | 0.14261 | 0.8015844 | 1.3606156 |
|  | 0.6 - 0.8 | 1.11593 | 0.27554 | 0.11249 | 0.8954496 | 1.3364104 |
|  | 1.1 - 1.8 | 1.04377 | 0.34216 | 0.13968 | 0.7699972 | 1.3175428 |
|  | 2-3 | 1.06573 | 0.25682 | 0.10485 | 0.860224 | 1.271236 |
|  | 50 | 1.29113 | 0.33012 | 0.13477 | 1.0269808 | 1.5552792 |
| Lactate | ≤ 0.5 | 1.40303 | 0.57845 | 0.23615 | 0.940176 | 1.865884 |
|  | 0.6 - 0.8 | 1.70063 | 0.65751 | 0.26843 | 1.1745072 | 2.2267528 |
|  | 1.1 - 1.8 | 1.7082 | 0.76397 | 0.31189 | 1.0968956 | 2.3195044 |
|  | 2-3 | 1.93413 | 0.89153 | 0.36396 | 1.2207684 | 2.6474916 |
|  | 50 | 1.26813 | 0.61486 | 0.25102 | 0.7761308 | 1.7601292 |
| Formiate | ≤ 0.5 | 14.27893 | 5.44744 | 2.22391 | 9.9200664 | 18.6377936 |
|  | 0.6 - 0.8 | 15.64993 | 5.57351 | 2.27537 | 11.1902048 | 20.1096552 |
|  | 1.1 - 1.8 | 14.57273 | 5.3802 | 2.19646 | 10.2676684 | 18.8777916 |
|  | 2-3 | 15.16483 | 5.43222 | 2.2177 | 10.818138 | 19.511522 |
|  | 50 | 11.59613 | 4.51575 | 1.84355 | 7.982772 | 15.209488 |
| Acetate | ≤ 0.5 | 20.2293 | 5.22345 | 2.13246 | 16.0496784 | 24.4089216 |
|  | 0.6 - 0.8 | 19.5867 | 4.57241 | 1.86668 | 15.9280072 | 23.2453928 |
|  | 1.1 - 1.8 | 19.6175 | 7.47534 | 3.0518 | 13.635972 | 25.599028 |
|  | 2-3 | 18.8414 | 5.27326 | 2.1528 | 14.621912 | 23.060888 |
|  | 50 | 21.00663 | 9.84474 | 4.0191 | 13.129194 | 28.884066 |
| Hydrogen | ≤ 0.5 | 3.23537 | 1.23943 | 0.50599 | 2.2436296 | 4.2271104 |
|  | 0.6 - 0.8 | 3.39433 | 1.24363 | 0.50771 | 2.3992184 | 4.3894416 |
|  | 1.1 - 1.8 | 3.12527 | 0.83986 | 0.34287 | 2.4532448 | 3.7972952 |
|  | 2-3 | 3.55627 | 0.98757 | 0.40317 | 2.7660568 | 4.3464832 |
|  | 50 | 2.4492 | 0.91816 | 0.37484 | 1.7145136 | 3.1838864 |
| Ethanol | ≤ 0.5 | 22.72843 | 1.55003 | 0.6328 | 21.488142 | 23.968718 |
|  | 0.6 - 0.8 | 23.1525 | 2.0715 | 0.84569 | 21.4949476 | 24.8100524 |
|  | 1.1 - 1.8 | 22.22543 | 0.86774 | 0.35425 | 21.5311 | 22.91976 |
|  | 2-3 | 22.1032 | 0.79597 | 0.32495 | 21.466298 | 22.740102 |
|  | 50 | 22.28167 | 1.84565 | 0.75348 | 20.8048492 | 23.7584908 |

**A5.2 Evaluation batch vs. fed batch**

Table S3: Mean values, standard deviation and standard error of the mean from the conducted tukey test for batch vs. fedbatch.

| Substrate | Feeding | Mean value | Standard deviation | Standard Error of the Mean | 95 % -Confidence  Intervals  Lower boundary | 95 % -Confidence  Intervals  Upper boundary |
| --- | --- | --- | --- | --- | --- | --- |
| Citrate | Batch | 0.05937 | 0.0084 | 0.00343 | 0.0526472 | 0.0660928 |
|  | FedBatch | 0.064 | 0.01363 | 0.00556 | 0.0531024 | 0.0748976 |
| Malate | Batch | 0.68327 | 0.3747 | 0.15297 | 0.3834488 | 0.9830912 |
|  | FedBatch | 0.4745 | 0.27246 | 0.11123 | 0.2564892 | 0.6925108 |
| Succinate | Batch | 1.0883 | 0.57664 | 0.23541 | 0.6268964 | 1.5497036 |
|  | FedBatch | 0.9346 | 0.40594 | 0.16572 | 0.6097888 | 1.2594112 |
| Lactate | Batch | 15.52047 | 8.42591 | 3.43986 | 8.7783444 | 22.2625956 |
|  | FedBatch | 10.91193 | 6.15546 | 2.51296 | 5.9865284 | 15.8373316 |
| Formiate | Batch | 24.79383 | 6.2265 | 2.54196 | 19.8115884 | 29.7760716 |
|  | FedBatch | 23.36633 | 5.81654 | 2.37459 | 18.7121336 | 28.0205264 |
| Acetate | Batch | 16.39443 | 3.14064 | 1.28216 | 13.8813964 | 18.9074636 |
|  | FedBatch | 17.0818 | 2.0993 | 0.85704 | 15.4020016 | 18.7615984 |
| Hydrogen | Batch | 4.32937 | 2.24023 | 0.91457 | 2.5368128 | 6.1219272 |
|  | FedBatch | 4.40713 | 1.61121 | 0.65777 | 3.1179008 | 5.6963592 |
| Ethanol | Batch | 14.7894 | 1.2527 | 0.51141 | 13.7870364 | 15.7917636 |
|  | FedBatch | 15.60677 | 2.21379 | 0.90377 | 13.8353808 | 17.3781592 |
